# Simulation of bright and dark diffuse multiple scattering lines in high-flux synchrotron X-ray experiments

**DOI:** 10.1107/S1600576725003553

**Published:** 2025-05-31

**Authors:** Maurício B. Estradiote, A. Gareth A. Nisbet, Rafaela F. S. Penacchio, Marcus A. R. Miranda, Guilherme A. Calligaris, Sérgio L. Morelhão

**Affiliations:** a Institute of Physics, University of São Paulo, São Paulo, SP, Brazil; b Diamond Light Source, Harwell Science & Innovation Campus Harwell OX11 0DE United Kingdom; c Institute of Exact and Natural Sciences Federal University for International Integration of the Afro-Brazilian Lusophony Redenção CE Brazil; d Brazilian Synchrotron Light Laboratory (LNLS) Brazilian Center for Research in Energy and Materials (CNPEM) Campinas SP Brazil; Tohoku University, Japan

**Keywords:** diffuse scattering, defects in crystals, synchrotron X-rays, multiple diffraction, structure properties

## Abstract

We present a theoretical framework for understanding diffuse multiple scattering (DMS) in single crystals, focusing on diffuse scattering Bragg channels. These channels, when probed with high-flux low-divergence monochromatic synchrotron X-rays, provide well defined visualizations of Bragg cones. Our main contribution lies in modelling the intensity distribution along these lines by considering diffuse scattering (DS) around individual reciprocal-lattice nodes. The model incorporates contributions from both general DS and mosaicity, elucidating their connection to second-order scattering events. This comprehensive approach advances our understanding of DMS phenomena, enabling their use as probes for complex material behaviour, particularly under extreme conditions.

## Abstract 2

This research introduces a theoretical framework for analysing diffuse multiple scattering in single crystals. Using high-intensity synchrotron X-rays, the model accurately predicts the intensity distribution along Bragg cones, taking into account both general diffuse scattering and mosaicity to understand complex material behaviour, especially in extreme environments.

## 1. Introduction

X-ray diffuse scattering (DS) in crystals arises from any deviation from perfect periodicity, encompassing phenomena such as atomic thermal vibrations, defects and local disorder (Debye, 1913 ▸; Waller, 1923 ▸; Woo, 1931 ▸; Zachariasen, 1940 ▸; Warren, 1990 ▸). Thermal DS contains information about lattice dynamics and, historically, it was the first tool utilized for experimental determination of phonon dispersion relations (Olmer, 1948 ▸; Cole & Warren, 1952 ▸; Joynson, 1954 ▸; Jacobsen, 1955 ▸).

The advent of synchrotron radiation technology has not only revitalized DS as a feasible probe for studying phonons (Holt *et al.*, 1999 ▸; Xu & Chiang, 2005 ▸; Mei *et al.*, 2015 ▸) but also transformed it into a powerful technique for investigating a wide range of order/disorder-related phenomena (Welberry, 2010 ▸; Barabash *et al.*, 2012 ▸; Kopecký *et al.*, 2012 ▸; Roth *et al.*, 2021 ▸; Holm *et al.*, 2021 ▸; Schmidt *et al.*, 2022 ▸; Takada *et al.*, 2022 ▸; Weadock *et al.*, 2023 ▸; Osborn, 2023 ▸; Guo *et al.*, 2023 ▸; Britt & Siwick, 2023 ▸; Subires *et al.*, 2023 ▸; Zacharias *et al.*, 2023 ▸). Synchrotron X-rays of very high flux density, approaching 10^15^ photons s^−1^ mm^−2^, have also revealed relatively un­known second-order scattering processes between DS sources and Bragg reflections. While the Bragg-DS channel produces nebulous and poorly localized intensity distributions, the DS-Bragg channel, where X-rays originating from DS undergo subsequent Bragg diffraction, yields well defined intensity lines (Ramsteiner *et al.*, 2009 ▸; Nisbet *et al.*, 2015 ▸). The diffuse multiple scattering (DMS) lines, as they have been called, are similar in appearance to pseudo-Kossel lines (Tixier & Waché, 1970 ▸; Morelhão & Cardoso, 1991 ▸; Bortel *et al.*, 2005 ▸) but are produced without using divergent-beam X-ray generators or any instrumental artefacts to provide divergent beams of monochromatic X-rays. Conversely, DMS lines become visible only with very narrow and highly parallel monochromatic beams, arising primarily from DS sources within the crystal.

Increasingly accessible with advanced X-ray sources and detectors, DMS lines offer unique insights into crystal defects, strain fields and lattice dynamics, complementing traditional diffraction techniques (Nisbet *et al.*, 2021 ▸; Nisbet *et al.*, 2023 ▸). The growing interest in utilizing DMS lines stems from their sensitivity to subtle crystallographic changes, coupled with the ability to monitor these changes across many directions simultaneously by analysing scattered X-rays within a single, relatively small, solid angle. This capability makes DMS lines a powerful probe in experiments with limited instrumental degrees of freedom, such as those conducted under extreme conditions. To exploit this potential fully, a comprehensive approach for structure modelling based on DMS lines is essential. While determining the geometric positions of these lines from the projection of Bragg cones (BCs) is relatively straightforward with existing software tools, like the *klines* module in the *PyDDT* package (Penacchio *et al.*, 2023 ▸), modelling the intensity distribution along the lines is challenging. This work introduces a theoretical framework to achieve this capability.

## 2. Theoretical framework

**Figure 1:**
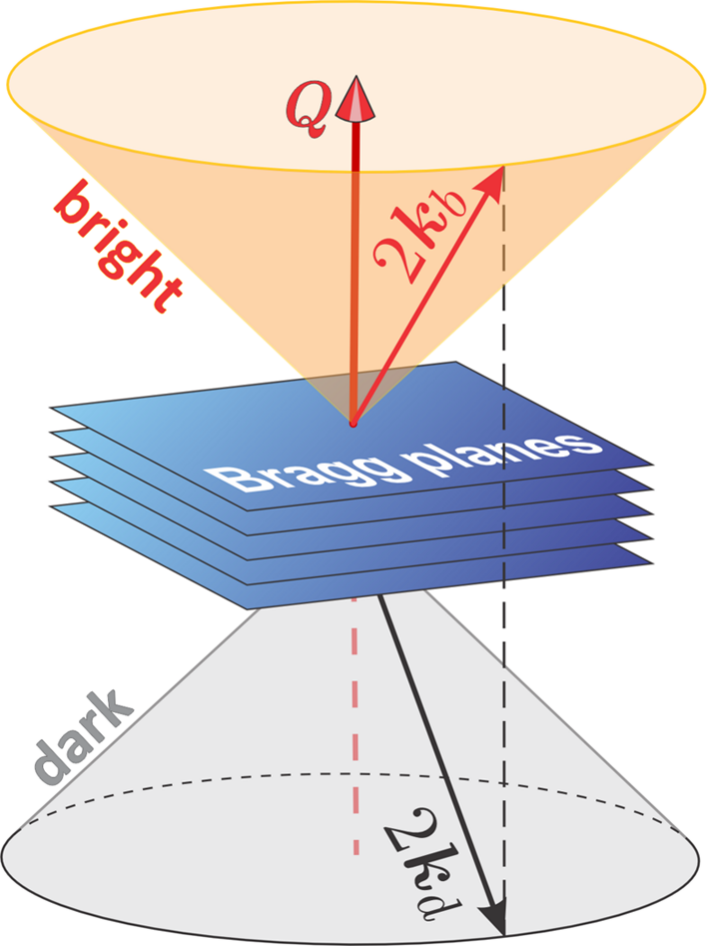
Bright and dark BCs for diffraction vector **Q**. X-rays with wavevector **k**_d_ propagating along the dark cone are attenuated by diffraction as they are scattered towards the bright cone with wavevector **k**_b_ = **Q** + **k**_d_.

One approach for accomplishing this task is to exploit the concept of BCs further, but distinguishing between two sets of wavevectors that are related to a reciprocal-lattice vector **Q** [*Q* = (4π/λ) sin(θ), where θ is half the scattering angle and λ is the wavelength of the incident radiation]. In Fig. 1 ▸ these two sets are depicted: the bright cone representing the set of wavevectors **k**_b_ that fulfil the condition 2**k**_b_ · **Q** = |**Q**|^2^, and the dark cone representing the set of wavevectors **k**_d_ that fulfil 2**k**_d_ · **Q** = −|**Q**|^2^, where **k**_b_ = **Q** + **k**_d_.

**Figure 2:**
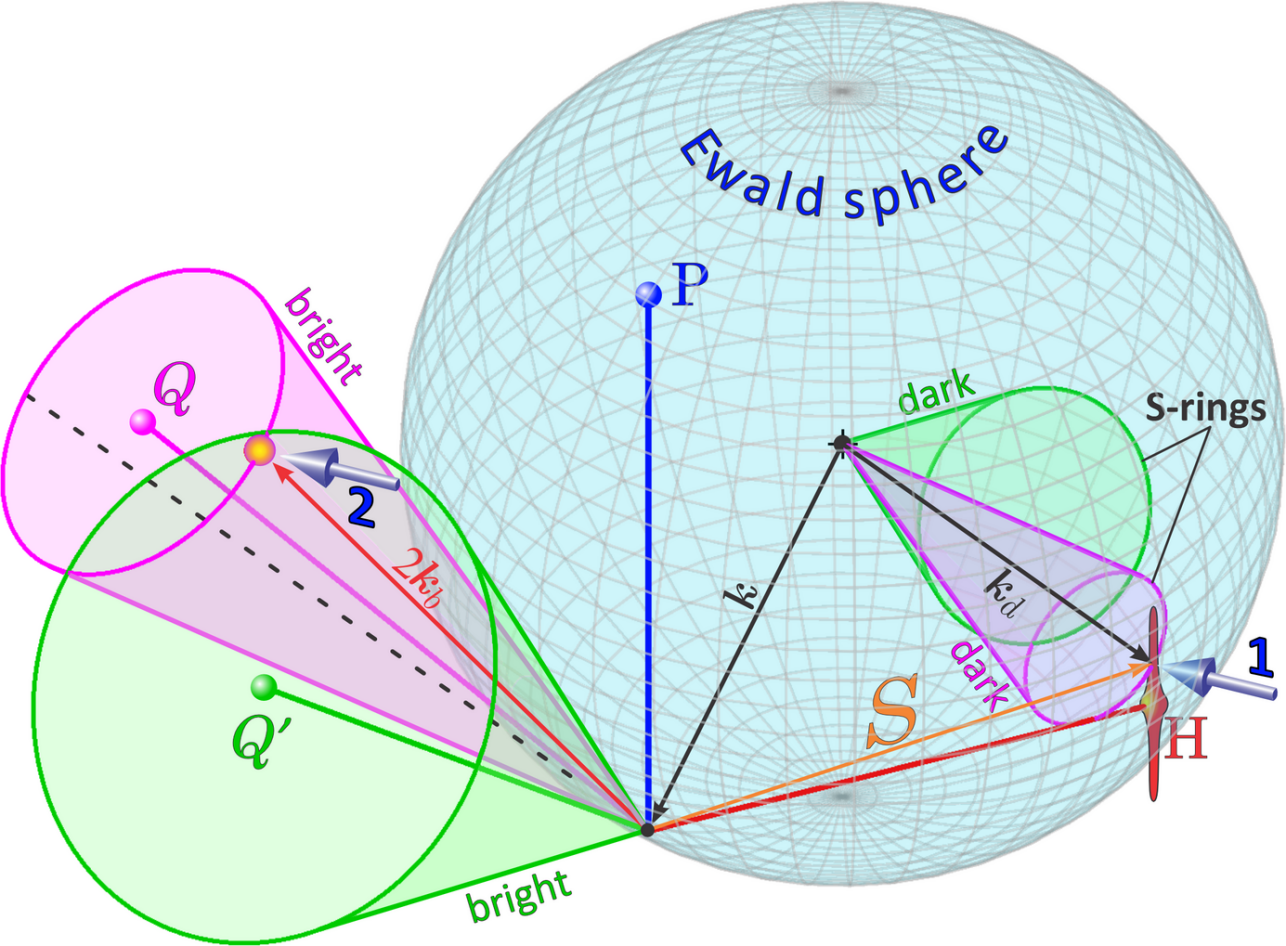
Modified Ewald construction to describe second-order scattering events giving rise to DMS lines. Bright cones have their apex at the reciprocal-space origin, while dark cones have their apex at the centre of the Ewald sphere, as shown for reflections *Q* and *Q*′. When scattering vectors of first order (**S** vectors) terminate at *S*-rings (dark cone–sphere intersections), the resulting second-order scattering is directed along the bright cones. For instance, the *S*-ring of reflection *Q* touches an intensity distribution near the reciprocal node of reflection *H* (arrow 1), defining the wavevector **k**_d_ = **S** + **k** that is re-scattered as **k**_b_ = **Q** + **k**_d_ along the *Q* bright cone (arrow 2); the **k**_d_ direction on the bright cone is indicated as a dashed line.

Fig. 2 ▸ shows the Ewald construction in reciprocal space, where dark cones have their apex at the centre of the Ewald sphere. The dark cones represent possible directions of wavevectors capable of undergoing Bragg diffraction, meaning these wavevectors terminate on the sphere’s surface. This also implies that the dark cones move as the direction of the incident wavevector **k** changes. The intersection of the dark cone with the surface of the Ewald sphere defines a ring containing all physically possible elastic scattering vectors **S** = **k**_d_ − **k** capable of providing an intensity contribution to the bright cone through diffraction vector **Q**. Bright cones have their apex fixed at the origin of reciprocal space, as their positions are independent of the incident beam direction. The intensity along a DMS line – the projection of the bright cone onto the detector area – is influenced by three key factors besides the X-ray polarization:

(i) Proximity of the *S*-ring (the set of scattering vectors **S**) to reciprocal-lattice nodes: closer proximity to a node results in higher intensity at specific points along the line.

(ii) Intensity distribution around each node: the unique intensity pattern surrounding each node creates peculiar features in the line.

(iii) Amount of DS between nodes: DS contributes to the overall visibility of the DMS line.

In the example of Fig. 2 ▸, the dark cone of reflection *Q* intercepts the Ewald sphere close to the node of reflection *H*. As the corresponding *S*-ring probes the node’s nearby intensity, an enhancement in intensity can be seen over the DMS line of reflection *Q*. When the *S*-ring touches the node, there is a vector **S** coinciding with the node’s reciprocal-lattice vector **H** and, in this case, multiple diffraction (MD) dynamical theory (Chang, 1984 ▸; Weckert & Hümmer, 1997 ▸; Authier, 2008 ▸) is necessary for a proper description of the intensities of the strongly coupled X-ray waves propagating inside the crystal: one wave from the reflection *H* and another from the reflection *P* = *H* + *Q*.

Aiming to treat DMS intensities that are visible away from strong Bragg reflections, the second-order solution of the MD (Shen *et al.*, 2000 ▸; Morelhão & Kycia, 2002 ▸; Shen *et al.*, 2006 ▸) provides a suitable approach for describing situations where the wavefield **D**_1_ of the *P* reflection, usually referred to as the primary reflection, is much weaker than the wavefield **D**_2_ from the sequence of *H* and *Q* reflections, also known as the *Umweg* wave (Stetsko *et al.*, 2000 ▸; Morelhão & Avanci, 2001 ▸). When the **S** vectors are away from any reciprocal-lattice node, the wavefield inside the crystal, **D** = **D**_1_ + **D**_2_, simplifies to
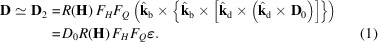
In standard MD treatments concerned with phase measurements by the interference between the **D**_1_ and **D**_2_ wavefields (Shen *et al.*, 2006 ▸; Morelhão *et al.*, 2011 ▸; Amirkhanyan *et al.*, 2014 ▸; Morelhão *et al.*, 2015 ▸; Morelhão *et al.*, 2017 ▸; Valério *et al.*, 2020 ▸; Penacchio *et al.*, 2023 ▸), the resonance term *R*(**H**) determines the excitation of the *Umweg* wave as a function of the *H* node distance to the surface of the Ewald sphere or, equivalently, to the *S*-ring. *F*_*X*_ stands for the structure factor of reflection *X* (= *H* or *Q*), and the directions 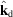 and 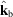 of the wavevectors on the dark and bright cones, respectively, of the *Q* reflection provide the polarization factor 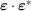, computed with respect to the state of polarization of the incident wave 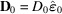.

An expression for simulating the intensity distribution along the DMS line of a given reflection *Q* follows directly from equation (1) as 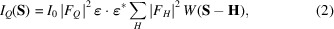
where the previous resonance term is replaced by a more general function *W*(**S** − **H**) to take into account elastic scattering that occurs away from exact Bragg conditions. Within this simple approach, the same function *W* applies to all *H* nodes and the DS intensities can be described as a discrete superposition of contributions from each individual *H* node. However, this approach can easily be extended to more complex situations that require a particular *W* function for each node to give a proper description of DS in the whole reciprocal space accessible by a given *S*-ring.

To compute the relative widths of observable DMS lines, the dynamic intrinsic width Λ_θ_ of the reflectivity curve in specular scattering geometry is used as a reference (Authier, 2008 ▸; Als-Nielsen & McMorrow, 2011 ▸; Morelhão, 2016 ▸). In reciprocal space, it is accounted for as a variation in the modulus *Q* of the diffraction vector, that is, *Q*^2^ → (*Q* ± Λ/2)^2^ ≃ *Q*^2^ ± *Q*Λ, where 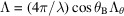 and θ_B_ is the Bragg angle of reflection *Q*. This results in a selection criterion for the full set of wavevectors on the bright BC, in which 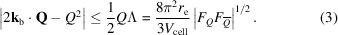
Here, *r*_e_ = 2.818 × 10^−5^ Å is the classical electron radius and *V*_cell_ is the unit-cell volume in Å^3^. In practice, equation (3) implies that the widths of DMS lines are proportional to the structure factor modulus of the corresponding *Q* reflection, as 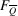 refers to the −*Q* reflection and 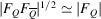. Note that the full set of wavevectors on the bright BC of reflection *Q* match, exactly, the set of wavevectors on the dark BC of reflection −*Q*. Consequently, when DS intensities from primary sources are directly measurable, dark BCs can become visible as shadows against such diffuse signals. In other words, the intensity along a DMS line can appear, in principle, as either positive or negative with respect to the existing scattered intensity in the detector area.

Simulating the intensity of DMS lines requires modelling the three-dimensional intensity distribution around each node. Besides general DS sources such as point defects, stacking faults, atomic thermal vibrations and many other types of deviation from the average periodic structure (Holt *et al.*, 1999 ▸; Xu & Chiang, 2005 ▸; Mei *et al.*, 2015 ▸; Roth *et al.*, 2021 ▸; Weadock *et al.*, 2023 ▸; Osborn, 2023 ▸), crystal truncation is a deviation from infinite periodicity and must be taken into account as extended scattering sources close to the nodes, in addition to DS sources. As a first approximation to identify experimental conditions favourable to the visibility of DMS lines, isotropic DS centred on each node and truncation rods in crystal slabs of outward surface normal direction 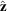 are modelled as follows. 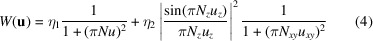
in equation (2) is written in terms of the distance 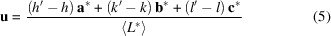
from each node, that is, from each reflection *H* of indices *hkl*. The non-integer indices of the scattering vectors **S** are obtained as described in Appendix *C*[App appc] or, in simpler terms, as *h*′ = **S** · **a**/2π, *k*′ = **S** · **b**/2π and *l*′ = **S** · **c**/2π through the unit-cell edge vectors **a**, **b** and **c**, while 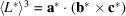 is the reciprocal unit-cell volume. The extent 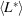/(π*N*) of isotropic DS from the nodes is adjustable by the number *N*. η_1_ and η_2_ weight the DS and crystal truncation rod (CTR) contributions in the case of a thin slab with a thickness of *N*_*z*_ unit cells of mean edge 2π/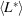. For slabs thicker than the beam coherence length and/or X-ray penetration depth, the sinc-squared function is replaceable by its enveloping Lorentzian function 1/[1 + (π*N*_*z*_*u*_*z*_)^2^] and *N*_*z*_ becomes an effective number, as the one in the last term regarding the effective in-plane slab dimension *N*_*xy*_ with *u*_*xy*_ = |**u** − **u**_*z*_| and 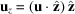. For nodes that are anisotropic regarding in-plane 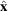 and 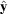 orthogonal directions, this last term gives way to other functions having, for instance, π*N*_*x*_*u*_*x*_ and π*N*_*y*_*u*_*y*_ as arguments where 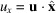 and 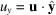.

## 3. Results and discussion

**Figure 3:**
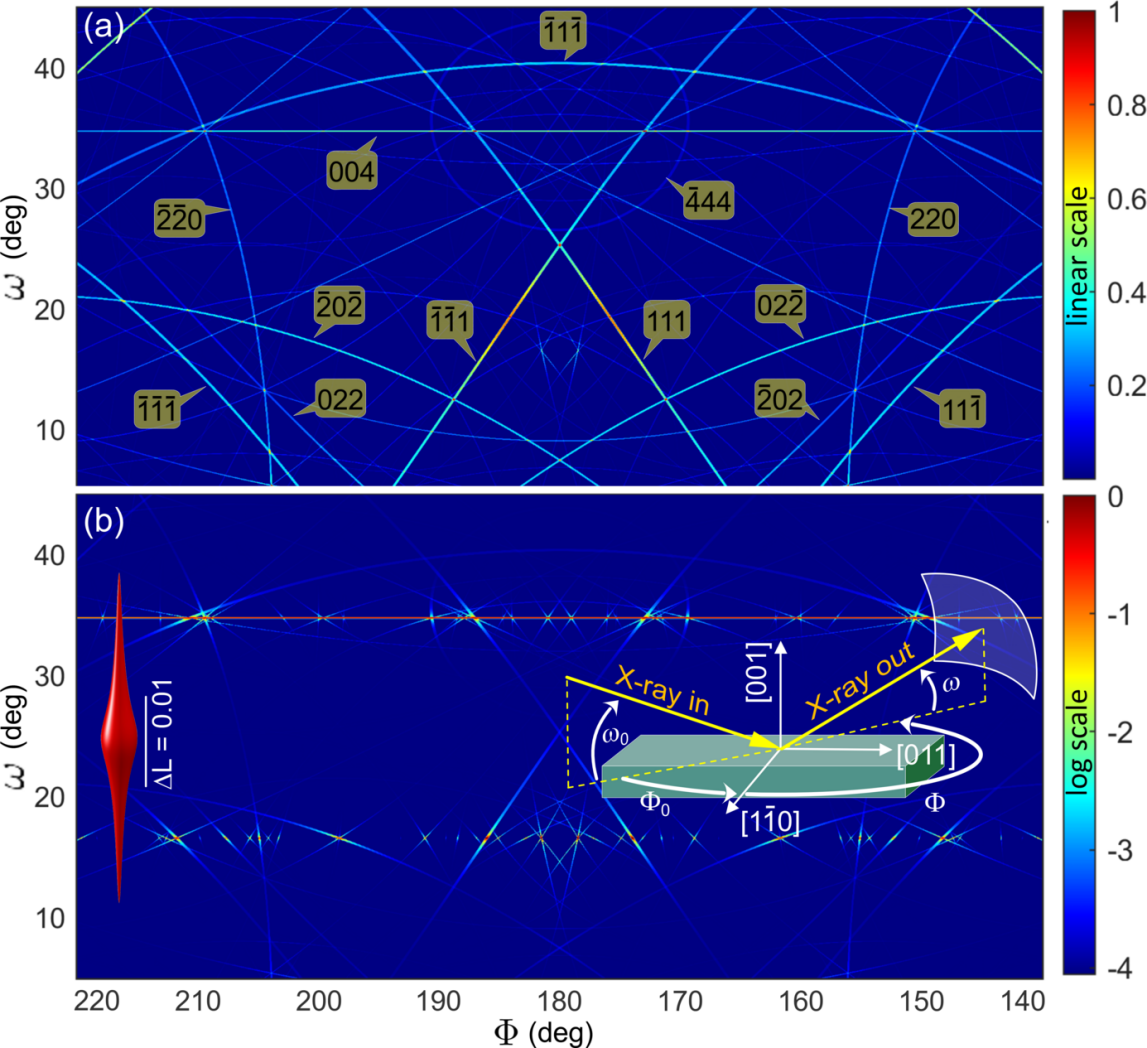
Simulation of bright DMS lines in Si(001) using equation (2). (*a*) Lines with smooth contrast variation due to the presence of an isotropic intensity distribution around each reciprocal node. Simulation parameters: η_1_ = 1, η_2_ = 0 and *N* = 1000 in *W*(**u**) [equation (4)]. Line indexing by *klines* (Penacchio *et al.*, 2023 ▸). (*b*) Lines with abrupt contrast variation due to an anisotropic intensity distribution around the reciprocal nodes. Simulation parameters: η_1_ = 0, η_2_ = 1, *N*_*xy*_ = 1000 and *N*_*z*_ = 100 in *W*(**u**), providing nodes elongated along the *L* reflection index (left inset, isosurface at 3% of the maximum). General simulation parameters: 8 keV σ-polarized X-rays, incidence direction with Φ_0_ = 0 and ω_0_ = 16.58° in the chosen reference frame (right-hand inset). Images are shown from the sample’s perspective (Φ values increasing from right to left) and with a resolution of 0.04° (pixel size).

Fig. 3 ▸ shows simulated DMS lines in silicon, where the line widths are proportional to |*F*_*Q*_| [equation (3)]. The incident beam direction is fixed at a chosen azimuth (Φ_0_) and incidence angle (ω_0_), ensuring that no allowed Bragg reflection is directly excited by the incident X-rays. This creates a null primary wavefield **D**_1_, a necessary condition for simulating DMS line intensities with equation (2) over wide solid angles.

In Fig. 3 ▸(*a*), the very smooth intensity variation of the DMS lines along their entire length results from the presence of DS intensities in all regions between reciprocal nodes. This is demonstrated using an isotropic DS centred on each node with a Lorentzian-like profile, that is, equation (4) with η_2_ = 0. In Fig. 3 ▸(*b*) a different situation is demonstrated, where intensity distributions in between reciprocal nodes are well pronounced along one direction only, that is, equation (4) with η_1_ = 0 and *N*_*z*_ << *N*_*xy*_; the appearance of these nodes is shown in Fig. 3 ▸(*b*) (left-hand inset). The anisotropy of the nodes creates a dynamic pattern of line contrast enhancement, shifting with changes in the incident beam direction (see animated GIFs in the supporting information).

**Figure 4:**
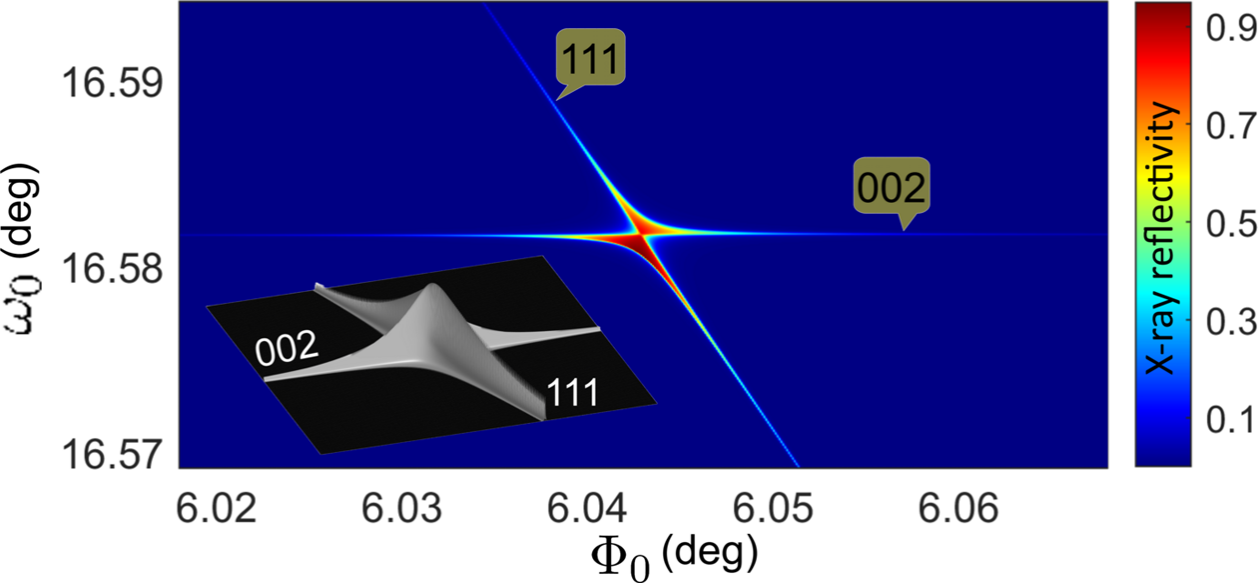
X-ray reflectivity of the ‘forbidden’ 002 reflection in silicon near the intersection of the 002 and 111 BCs, as a function of the incident beam direction (Φ_0_ and ω_0_ angles). Dynamical diffraction theory was applied for X-rays of 8 keV in a 100 µm thick Si(001) slab. (Inset) A 3D view of the reflectivity line profiles (on a linear scale) along the 002 and 111 BCs. Experimental data are reported elsewhere (Morelhão *et al.*, 2002 ▸; Domagała *et al.*, 2016 ▸).

Because the intensities at DMS line intersections come from uncorrelated DS sources at different reciprocal-space locations, as implicit in the deduction of equation (2), only intensity superposition is expected, without interference effects. Observing DMS lines and their intersections on the detector area requires a fixed incident beam; conversely, modulating the reflectivity of individual reflections is achieved by scanning the incident beam across the intersection of BCs (Morelhão & Cardoso, 1996 ▸; Hayashi *et al.*, 1997 ▸; Avanci *et al.*, 1998 ▸; Avanci *et al.*, 1999 ▸). For instance, the reflectivity of the ‘forbidden’ 002 reflection in silicon is observable only near BC intersections, as demonstrated in Fig. 4 ▸ through *n*-beam dynamical theory calculations for a thick crystal slab (Colella, 1974 ▸; Shen, 1993 ▸).

Observation of long DMS lines in perfect crystals is possible due to DS from thermal vibrations (Holt *et al.*, 1999 ▸), as in materials of high thermal conductivity such as copper. X-ray beams of high flux density are also necessary to observe DMS lines, such as the one available on beamline I16 at the Diamond Light Source: an in-vacuum undulator as the beamline insertion device, photon flux (beam intensity) above 10^12^ photons s^−1^ at 7.8 kev, energy resolution of a Si(111) double-crystal monochromator, beam size of 35 (vertical) × 184 (horizontal) µm and divergence of 0.04 (vertical) × 0.11 (horizontal) mrad (Collins *et al.*, 2010 ▸). Minimizing background noise in the detector area, such as that from air gaps in the beam path, helps enhance the contrast of the lines.

**Figure 5:**
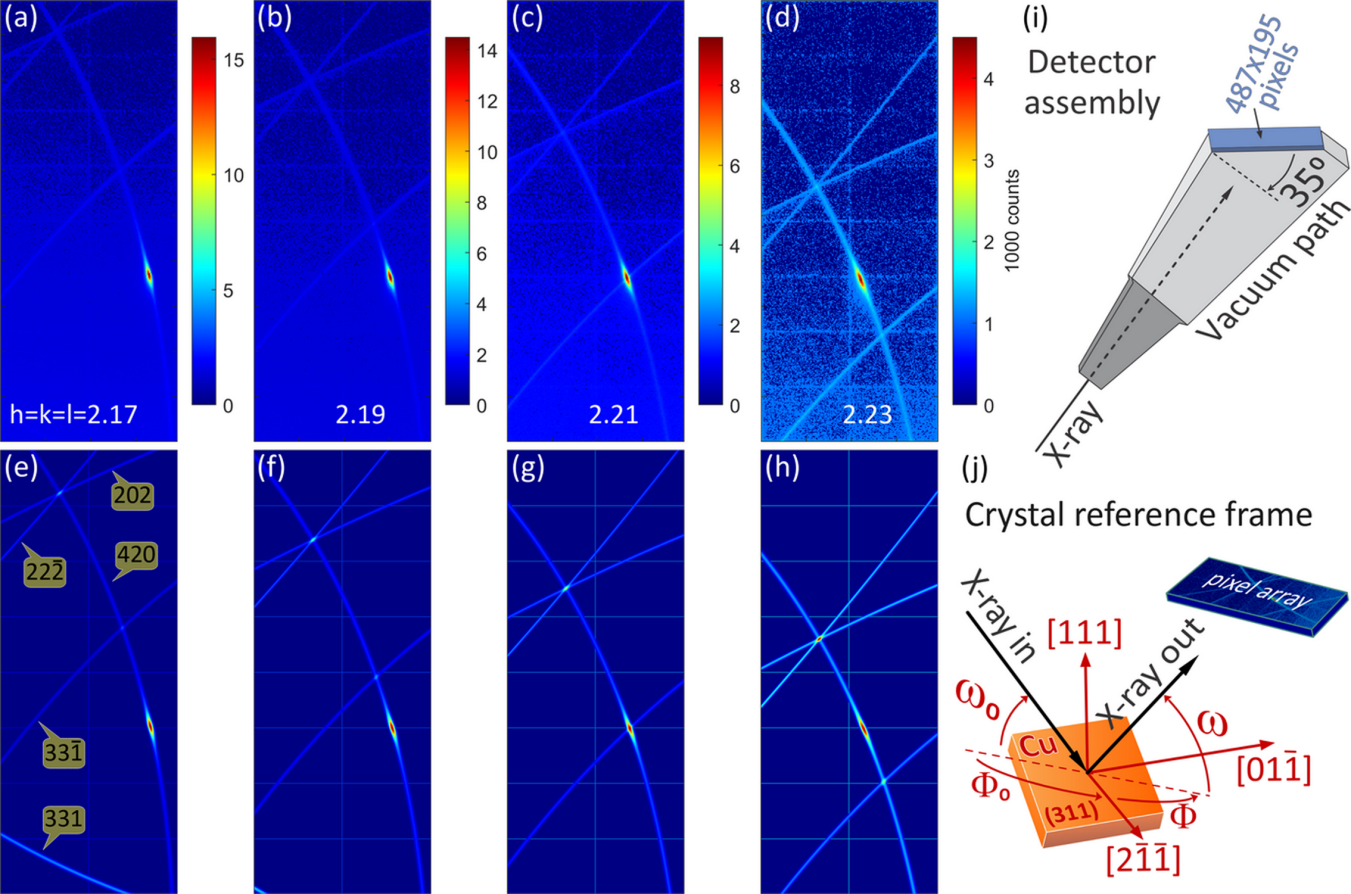
(*a*)–(*d*) Experimental observations and (*e*)–(*h*) simulations of DMS lines in a Cu(311) single crystal with high-flux synchrotron X-rays of 7.82 keV (λ = 1.585486 Å), σ-polarization (vertical scattering plane). (*i*) Detector assembly with pixel array rotated by 35° and centred at 565 mm from the sample, Diamond Light Source beamline I16 (Collins *et al.*, 2010 ▸), Pilatus 100k detector (487 × 195 pixels of 0.172 mm). (*j*) Crystal reference frame. ω_0_ and ω (detector’s central pixel) are both equal to the Bragg angle θ_*hkl*_ with *h* = *k* = *l* in the range from 2.17 to 2.23, as indicated in the top panels. Φ_0_ = −134.56° and Φ = Φ_0_ + 180° in all cases. DMS line indices are shown in the bottom left-hand panel. Simulations are based on isotropic diffuse scattering to illuminate the DMS lines plus mosaicity at reflection 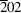 to account for the behaviour of the intensity spot (red spot) on the 420 DMS line (more images available in the supporting information).

Figs. 5 ▸(*a*)–5 ▸(*d*) and Fig. 7(*a*) show experimental DMS lines in a Cu(311) single crystal obtained on either vertical or horizontal scattering planes, σ- or π-polarization, respectively; see Appendix *A*[App appa] for the solid angles recorded on each scattering geometry in comparison with DMS lines on a wide solid angle. While most of the lines are easily simulated within the isotropic DS model, there are a few intensity features demanding more complex models than can be accomplished by the DS or CTR models in equation (4), in particular the well defined intensity spot on the 420 DMS line [Figs. 5 ▸(*a*)–5 ▸(*d*)] that is observed to move along the [111] direction, exactly as expected for a CTR in θ/2θ scans of symmetrical reflections (specular reflection geometry).

However, the direction normal to the sample surface is the [311] direction rather than [111], as depicted in Fig. 5 ▸(*j*), and this intensity spot moves in the opposite sense to what was predicted by our simulation when specifically accounting for CTR effects (animated GIFs in the supporting information).

**Figure 6:**
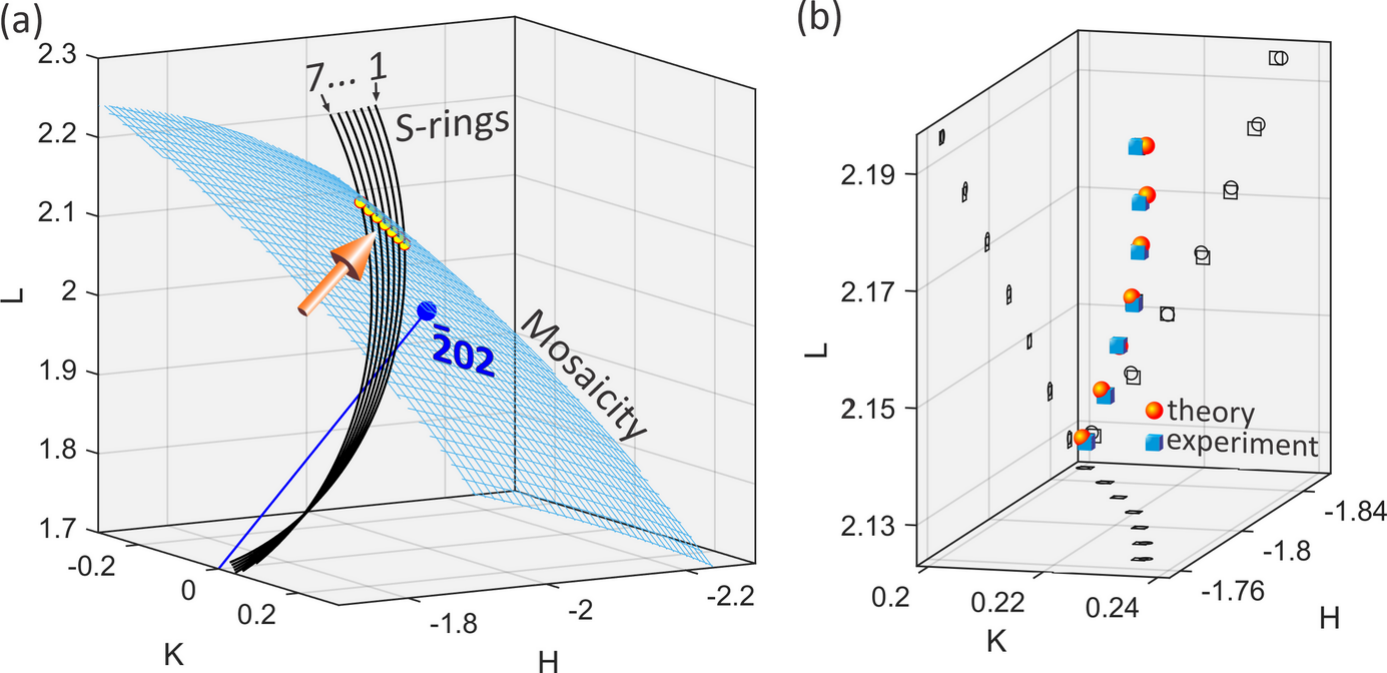
(*a*) Three surface intersection spots (arrow) between the 420 dark cone and Ewald sphere (*S*-rings) and a spherical shell of radius 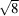 (mesh surface) standing for mosaicity around the 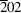 node. *S*-rings 1 to 7 correspond to changes in the incidence angle ω_0_ = θ_*hkl*_ with *h* = *k* = *l* varying from 2.17 to 2.23 in steps of 0.01, Φ_0_ = −134.56° and Φ = Φ_0_ + 180°. (*b*) Theoretical (red spheres) and experimental (blue cubes) intersection spots of the *S*-rings with the shell of grain misorientation. Projections of the interception points on the *HK*, *HL* and *KL* planes (open circles and open rectangles in dark grey) are also shown.

After each pixel of the detector area has been transformed, *e.g.* in Fig. 5 ▸(*e*), onto *hkl* coordinates around the 420 *S*-ring, the scattering vector responsible for the spot intensity ends at coordinates like *h*′ = −1.7988, *k*′ = 0.2246 and *l*′ = 2.1703 on the *S*-ring, whose modulus squared is very close to 8. The experimental data were then exactly simulated in Figs. 5 ▸(*e*)–5 ▸(*h*) by taking the source of spot intensity as located at the intersection between the 420 *S*-ring and a sphere of radius 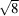, as shown in Fig. 6 ▸(*a*). Perfect agreement between the experimental and theoretical sets of *hkl* coordinates is demonstrated in Fig. 6 ▸(*b*), where the former set was obtained from the central pixel of the spot in each experimental image according to (see also Appendix *C*[App appc])
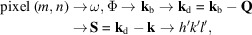
while the latter set was calculated by the intersection of three surfaces:
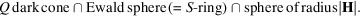
**Q** and **H** stand for diffraction vectors of reflections 420 and 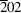, respectively. The shape and relative intensity of the spot were adjusted by adding to *W*(**u**) in equation (4) the term 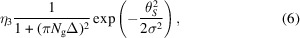
where Δ = (|**S**| − |**H**|)/|**H**| and θ_*S*_ is the angle between the **S**and **H** vectors, that is, Δ = 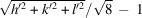. 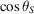
 = 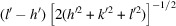, as copper is a cubic crystal. The simulations in Figs. 5 ▸(*e*)–5 ▸(*h*) were obtained using η_1_ = 1, *N* = 4/π, η_2_ = 0, η_3_ = 40, a grain size *N*_g_ = 242 Bragg planes (31 nm) and a mosaic spread σ = 3.2°. As the grain size is the only parameter determining the spot shape along the DMS line, it is the most accurate value with an uncertainty of about 20%. The other parameter values are related to each other and are therefore adjustable within wide ranges capable of generating similar images. Gaussian mosaicity was assumed for simplicity, but the actual sample mosaicity is far from Gaussian, as discussed in Appendix *B*[App appb].

**Figure 7:**
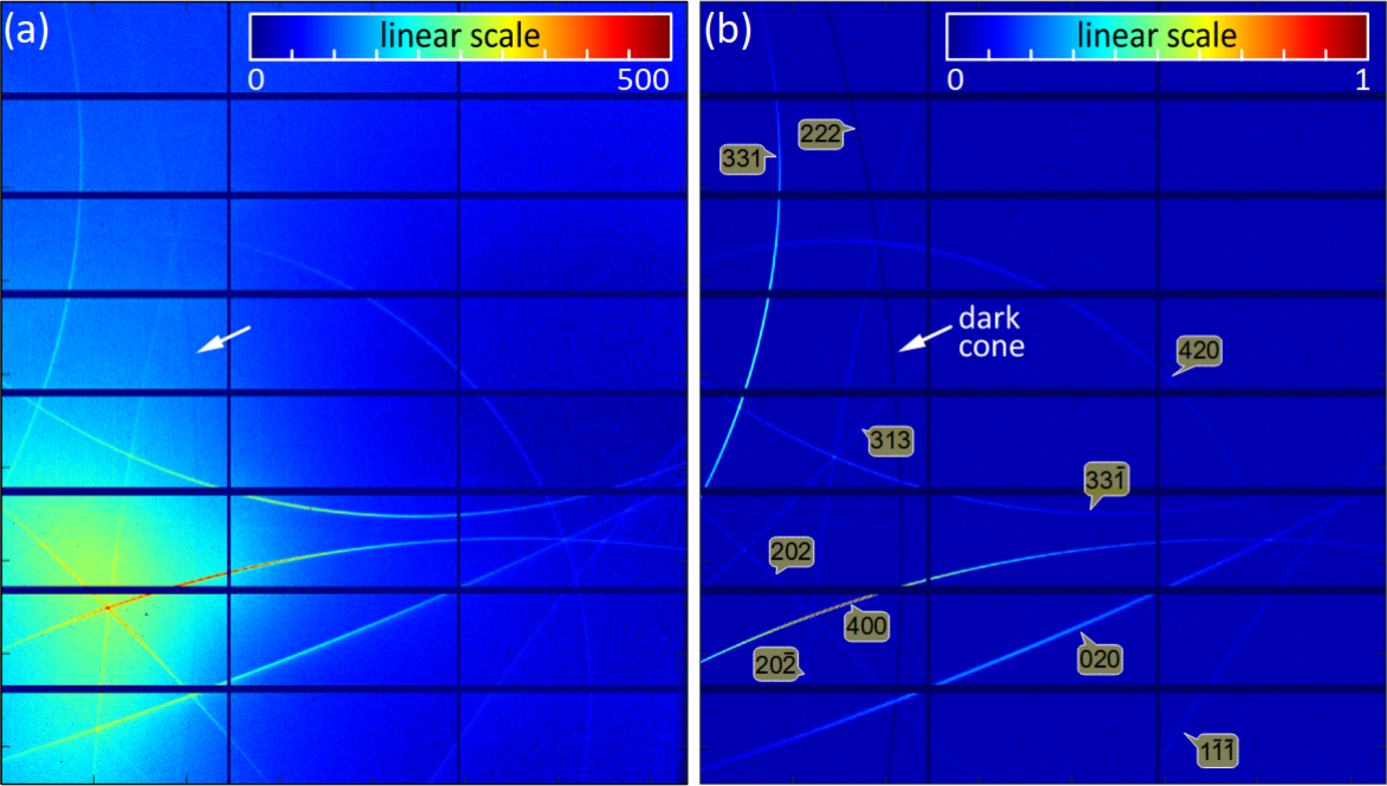
(*a*) Experimental observation and (*b*) simulation of DMS lines in a Cu(311) single crystal with high-flux synchrotron X-rays of 7.82 keV, π-polarization (horizontal scattering plane). A dark cone is also visible in the imaged area (white arrows). Detector area perpendicular and centred at 850 mm from the sample, Diamond Light Source I16 (Collins *et al.*, 2010 ▸), Pilatus 2M detector (1679 × 1475 pixels of 0.172 mm). ω_0_ = ω (pixel *m*_0_*n*_0_) = 45°, corresponding to *h* = *k* = *l* = 1.8618, Φ_0_ = 216° and Φ (pixel *m*_0_*n*_0_) = Φ_0_ − 180° in the reference frame of Fig. 5(*j*). Pixel *m*_0_*n*_0_ is defined in Appendix *A*1. Simulation based on a short-range DS model plus mosaicity; see text for details.

Fig. 7 ▸(*a*) shows the actual lengths of DMS lines tracked on a wide-area detector in horizontal scattering geometry (π-polarization). By adjusting the scattering angle to 2θ = ω_0_ + ω ≃ 90° (χ = 0, 2θ_d_ = 0 and φ_d_ ≃ 90° in Appendix *A*[App appa]), X-ray photons from single scattering events are strongly suppressed by polarization near the centre of the detection area, enhancing the visibility of DMS lines. Most of the lines are much shorter than expected within the long-range isotropic DS model in equation (4). A short-range model, obtained by exchanging 1/[1 + (π*Nu*)^2^] for 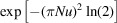 in equation (4), was used instead for better reproduction of the length of the lines. According to the theoretical approach in equation (2), the 400 line is expected to be 40% weaker than the 331 line and 80% weaker than the 020 line when considering only the nearest nodes of the respective dark cones as 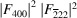 ≃ 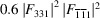 ≃ 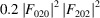. Simulation of this extra intensity of the 400 line was accomplished by adding mosaicity to the 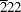 node, as in equation (6). For the other nodes, mosaicity contributions appear further away from the imaged area. η_1_ = 1, *N* = 5/π, η_2_ = 0, η_3_ = 2, θ_*S*_ = 0 and *N*_g_ = 11 were the parameter values used for the simulation shown in Fig. 7 ▸(*b*). A random value between zero and 0.1 was added as statistical noise to all pixels, except for those at the position of the 222 line (white arrow).

**Figure 8:**
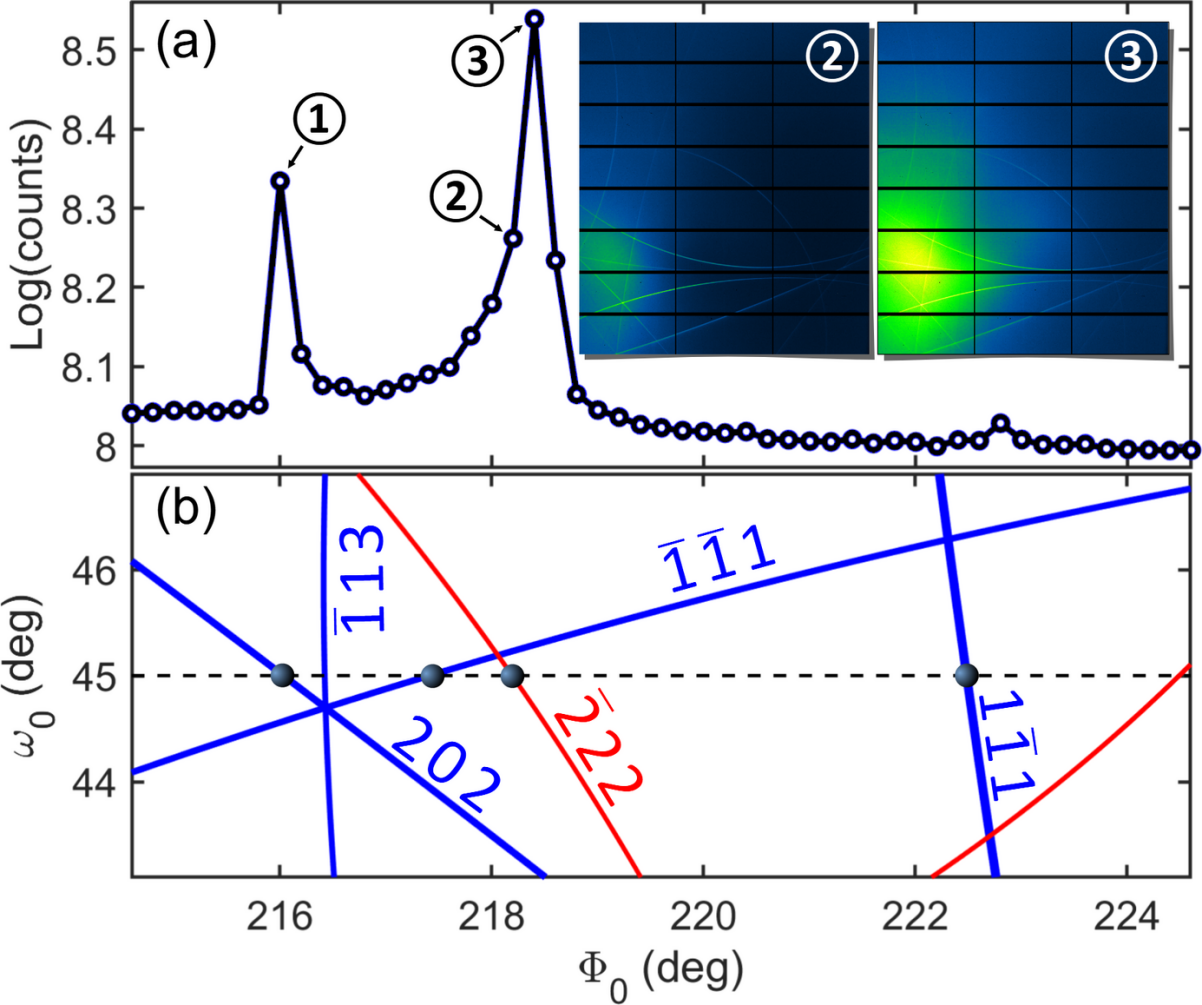
(*a*) Integrated detector intensity versus sample azimuthal angle Φ_0_ around [111]. Images at selected azimuths (arrows 2 and 3) are shown as insets and in Fig. 7(*a*) (arrow 1). (*b*) A 2D representation of Bragg cones versus incidence angles ω_0_ and Φ_0_. Intersections with a fixed value of ω_0_ = 45° (dashed line) indicate azimuths where Bragg-DS channels are excited.

The 222 bright cone, devoid of intensity contributions from DS sources, allows the 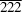 dark cone (white arrow) to become visible as a shadow in areas detecting nebulous scattering from Bragg-DS channels. In Fig. 8 ▸, Bragg reflections involved in these channels are identified by plotting the total integrated intensity across the entire detector area as a function of the sample’s azimuth, in comparison to a 2D graphic representation of Bragg cones as a function of incidence angles ω_0_ and Φ_0_. A stronger Bragg-DS channel, distinct from the one in Fig. 7 ▸(*a*) (caused by the 202 Bragg reflection), is excited at a slightly different azimuth, Φ_0_ = 218.4° (inset of Fig. 8 ▸). Observation of these second-order scattering channels provides further evidence of DS and/or mosaicity around reciprocal-lattice nodes. Their images represent the inter­section of the Ewald sphere associated with the Bragg-diffracted beam and the distribution of intensity in reciprocal space, analogous to the case of the incident X-ray beam’s Ewald sphere. However, Bragg-DS channels exhibit a strong dependence on azimuth, limiting their utility in 3D reciprocal-space mapping applications.

## 4. Conclusions

In conclusion, this work has introduced a robust theoretical framework for simulating X-ray diffuse multiple scattering lines, filling a significant gap in existing methodologies and providing a valuable tool for the detailed analysis of experimental data. The successful application to both silicon and copper single crystals, accounting for isotropic diffuse scattering and crystal truncation, demonstrates the broad applicability of this approach. The inclusion of grain misorientation effects highlights the potential of this framework to elucidate complex microstructural features. This work opens a new avenue for investigating diffuse scattering phenomena and their relationship to material properties, with the promise of furthering our understanding of crystal defects and their influence on a material’s behaviour.

## Supplementary Material

Supporting information file. DOI: 10.1107/S1600576725003553/jo5124sup1.pdf

Animated demonstration of CTR contributions to DMS line intensities in a Si(001) crystal. σ-polarization. DOI: 10.1107/S1600576725003553/jo5124sup2.gif

Animated demonstration of CTR contributions to DMS line intensities in a Si(001) crystal. π-polarization. DOI: 10.1107/S1600576725003553/jo5124sup3.gif

Animated demonstration of CTR contributions to DMS line intensities in a Cu(311) crystal. DOI: 10.1107/S1600576725003553/jo5124sup4.gif

## Figures and Tables

**Figure 9:**
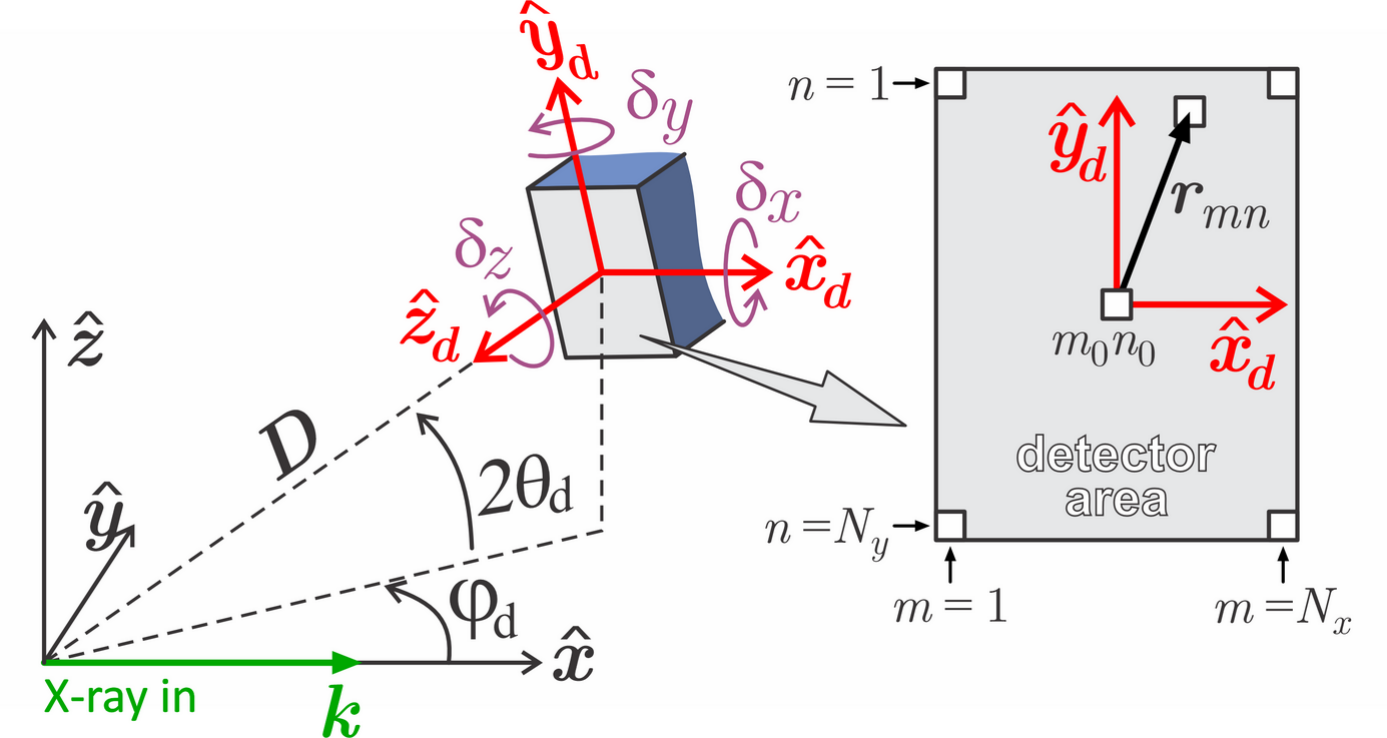
Laboratory reference frame 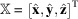 with the incident wavevector 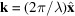 along the *x* axis. 2θ_d_ and φ_d_ are the detector arm elevation and azimuthal angle, respectively. The pixel of indices *m*_0_*n*_0_ is the one hit by the direct beam when 2θ_d_ = φ_d_ = 0. Tilt angles δ_*x*_, δ_*y*_ and δ_*z*_ are required for a general orientation of the pixel array, as in equation (9) where 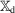 = 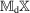 = 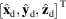.

**Figure 10:**
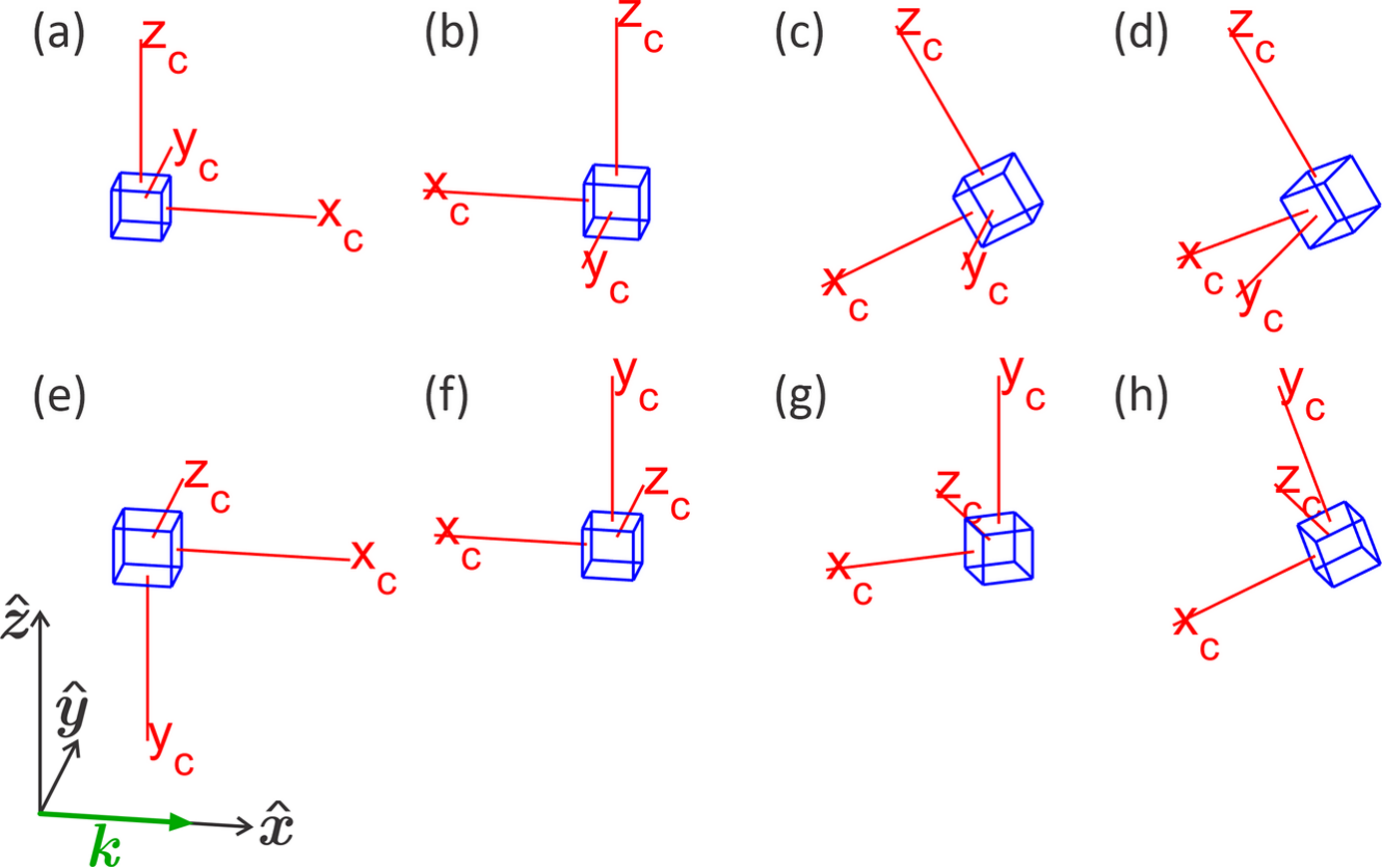
Orientation of the crystal reference frame 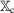 = 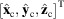 = 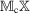 with respect to the laboratory frame 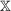 = 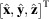. (*a*)–(*d*) Vertical scattering plane (*xz* plane), χ = 90°. (*e*)–(*h*) Horizontal scattering plane (*xy* plane), χ = 0. (*b*) and (*f*) Setting 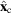 pointing to the X-ray source. (*c*) and (*g*) Counter-clockwise ω_0_ = 20° rotation around 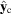. (*d*) and (*h*) Clockwise Φ_0_ = 25° rotation around 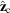.

**Figure 11:**
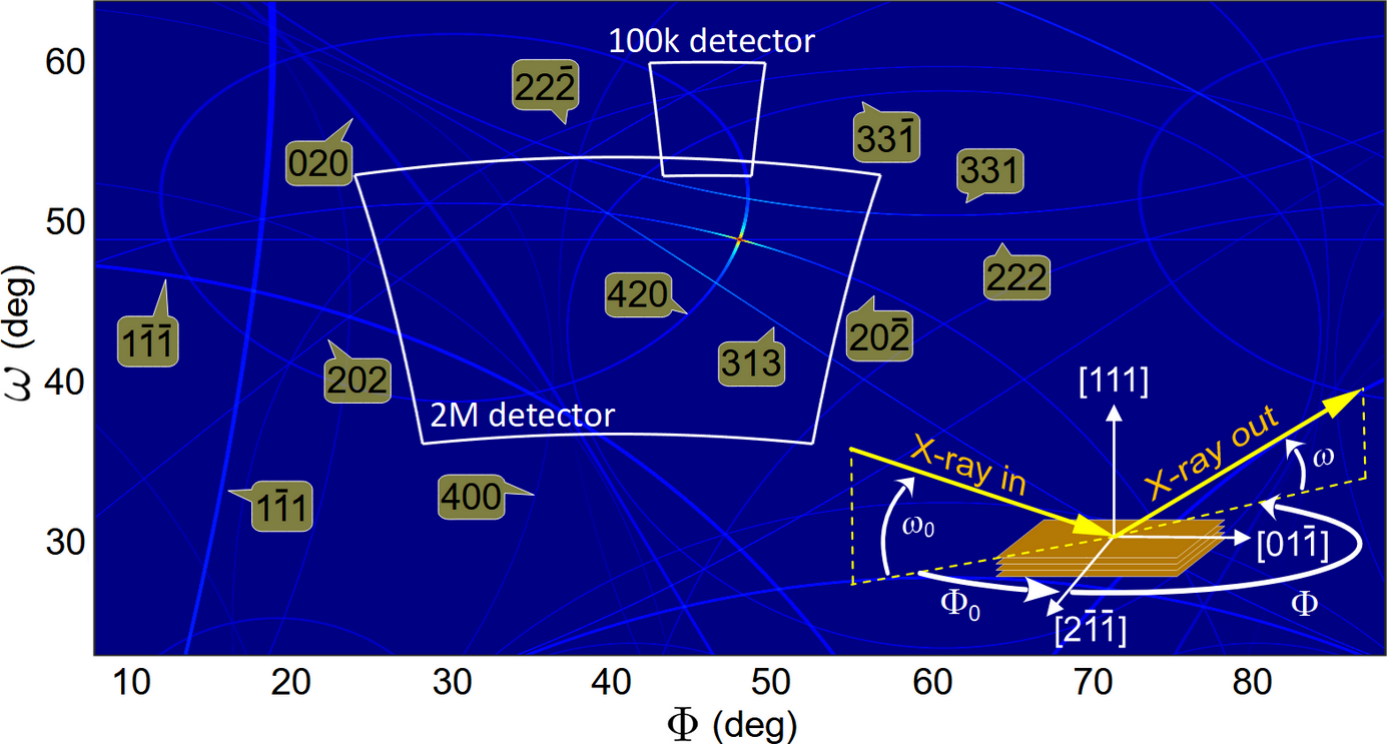
Simulation of bright DMS lines in Cu. Isotropic DS model for 7.82 keV X-rays, σ-polarization (χ = 90°). Incidence direction ω_0_ = 49.435° (Bragg angle of reflection 222) and Φ_0_ = 227.5° (multiple diffraction 222/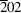) in the chosen crystal reference frame (inset), where **A** ∥ [111] and **B** ∥ [100] in equation (11). Line contrast is on a logarithmic scale. Solid angles are as observed by the arrays of the 100k (*N*_*y*_ = 487, *N*_*x*_ = 195) and 2M (*N*_*y*_ = 1679, *N*_*x*_ = 1475) detector pixels are indicated (white-outlined areas). For the 100k detector, *P* = [565 mm, 90°, 56.683°, 225.5°, 113.365°, 0, 35°, 0, 0]. For the 2M detector, *P* = [850 mm, 0,45°, 220°, 0, 91°, 0, 0, 0].

**Figure 12:**
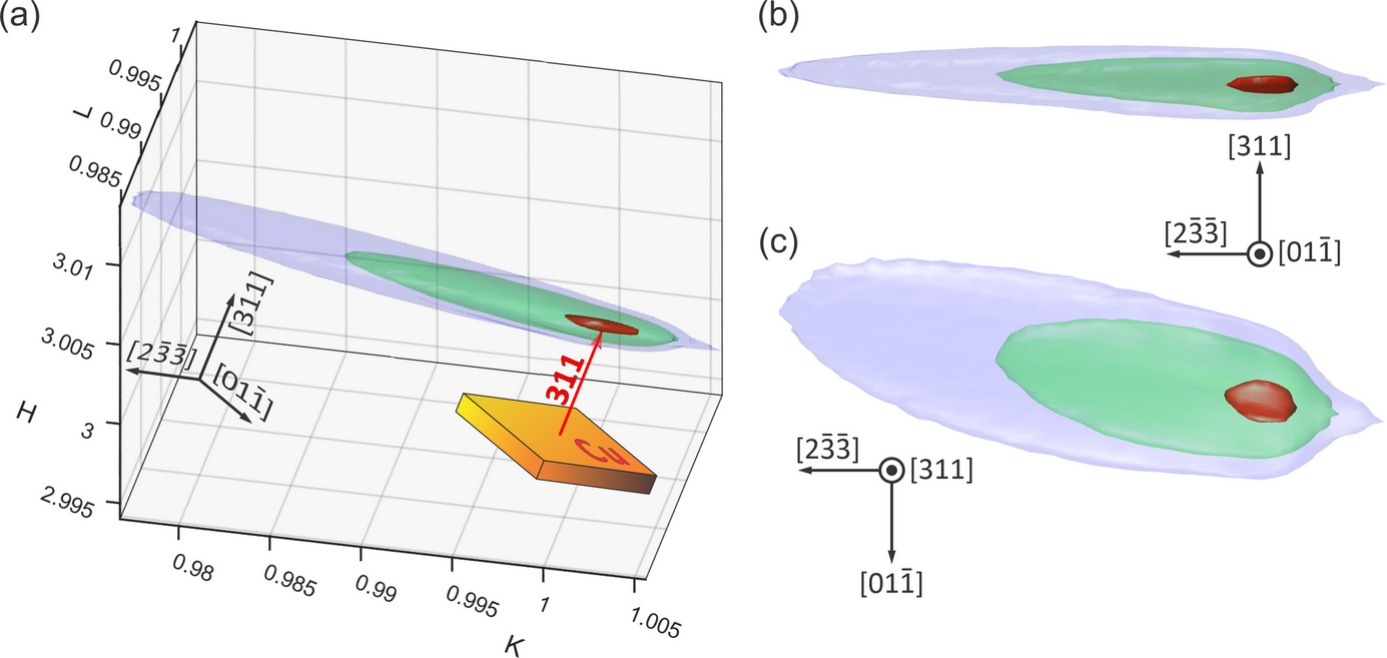
A 3D-RSM around the Cu(311) reflection, acquired using 8.0436 keV X-rays. The map is composed of 44 images with varying ω_0_ values, collected using a 100k detector with parameter vector *P* = [565 mm, 90°, ω_0_, 80.727°, 89.965, 0, 35°, 0, 0]. Crystal frame, **A** ∥ [311] and **B** ∥ [100]. Isosurfaces on a logarithmic scale are shown at 50% (red), 35% (green) and 30% (blue) of the log-transformed maximum intensity. (*a*) *hkl* coordinates, 311 diffraction vector (red arrow) not to scale. (*b*) Side view along the [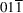] in-plane direction. (*c*) Top view along [311].

## Appendix A Reference frames for area detectors and single crystals

### A1. Detector reference frame

The laboratory reference frame of the synchrotron diffractometer is defined as follows (Penacchio *et al.*, 2022 ▸): the *x* axis is parallel to the incoming X-ray beam, pointing downstream; the *y* axis is horizontal and perpendicular to the *x* axis; and the *z* axis is vertical, perpendicular to both the *x* and *y* axes. This configuration forms a right-handed Cartesian coordinate system in which the detector central pixel, of indices *m*_0_*n*_0_, is placed at

where *D* is the sample-to-detector distance and  = ; hereinafter the superscript T stands for a transposed matrix. On the detector area, an array of *N*_*y*_ rows and *N*_*x*_ columns of pixels of size *p*, the position of each pixel is given by

with *m* = 1, 2,…*N*_*x*_ varying from left to right and *n* = 1, 2,…*N*_*y*_ varying from top to bottom, as shown in Fig. 9 ▸. When accounting for possible tilts and rotations of the detector area, its reference frame in terms of the laboratory frame becomes

where ,  and  are right-handed rotation matrices. δ_*x*_, δ_*y*_ and δ_*z*_ tilt the pixel array around the central row (direction ), central column (direction ) and its normal direction (direction ), respectively. Substituting  in equation (8), the 3D coordinates of the pixels in the laboratory frame are then computed as

### A2. Crystal reference frame

By choosing two non-collinear reciprocal vectors, such as **A** and **B**, a Cartesian reference frame for the crystal is built as follows,

where  is any Cartesian system upon which the reciprocal base vectors , ,  were defined. A reciprocal-lattice vector such as

is projected in this new crystal reference frame as  =  = . As  and  are Cartesian systems,  is a rotation matrix and  is the identity matrix.

The orientation of the crystal in the laboratory frame is based on three rotation angles: χ as the rotation of the scattering plane around the *x* axis, measured from the *xy* plane; ω_0_ as the incidence angle for which ∠(**k**, **A**) = 90° + ω_0_; and Φ_0_ as the angle of azimuthal rotation around vector **A**. As depicted in Fig. 10 ▸, we choose  pointing to the X-ray source when ω_0_ = Φ_0_ = 0, implying the following rotation matrix to orient the crystal in the laboratory frame:

In this frame, the incident wavevector **k** =  =  is written as

From equation (10), X-rays hitting the pixel array have wavevectors

that are projected as

providing the ω and Φ angles on which each pixel is seen in the crystal frame. The entire solid angle monitored by an area detector is then obtained as a function of nine degrees of freedom (one translational and eight rotational): sample-to-detector distance (*D*), sample rotation angles (χ, ω_0_ and Φ_0_), detector arm rotation angles (2θ_d_ and φ_d_) and tilt angles of the detector area (δ_*x*_, δ_*y*_ and δ_*z*_). This is summarized in a parameter vector *P* = [*D*, χ, ω_0_, Φ_0_, 2θ_d_, φ_d_, δ_*x*_, δ_*y*_, δ_*z*_] used as input to a computer program that provides matrices of ω and Φ angles from a pixel array. As an example, Fig. 11 ▸ compares the solid angles monitored by actual area detectors with the expected DMS lines of a Cu crystal.

## Appendix B Three-dimensional reciprocal-space map (3D-RSM)

A more general version of the simple recipe for generating 3D-RSMs (Penacchio *et al.*, 2022 ▸) is obtained by using the reference systems described above. For a given detector pixel, the diffraction vector

in the crystal’s Cartesian frame is readily projected, through the change of reference  =  [equation (11)], into the crystal’s reciprocal space as

or in *hkl* coordinates

given that  =  as defined in equation (12).

A 3D-RSM of the Cu(311) sample, shown in Fig. 12 ▸, reveals pronounced mosaicity preferentially oriented along one in-plane direction. This mosaicity primarily affects the intensities below the half-width of the diffraction peak, being about 0.5° at 30% of the log-transformed maximum intensity on its preferential direction and much wider at lower intensities. Its behaviour deviates significantly from a Gaussian mosaicity, in which the expected relative separation between the 30% and 50% isosurfaces (logarithmic scale) is only 1.76. As shown in Fig. 12 ▸(*c*), the observed separation between these isosurfaces is substantially larger, ranging from approximately 2.5 to 11. The surface finishing processes applied to the sample are the likely cause, introducing defects in a thin layer beneath the surface. The absence of crystal truncation rods, that is, an enhanced intensity along the [311] surface normal direction, is further evidence of poor surface crystalline quality.

## Appendix C Computational procedure

For simulating DMS lines, the following procedure has been used. Within the set of wavevectors  collected by the pixel array [equation (16)], those satisfying the bright cone equation

for the reciprocal-lattice vector **Q**^(c)^ are used to compute the corresponding wavevectors  =  on the dark cone, as well as the scattering vectors  =  =  of the *S*-ring. The intensity *I*_*Q*_(**S**) [equation (2)] of the DMS line for reflection *Q* is then computed either as a function of reciprocal-space coordinates,

or *hkl* coordinates,

of the scattering vectors. See the supporting information for details of how to adjust the line sharpness.

For the DMS line simulation in Fig. 11 ▸, we have used

where *r*_e_ = 2.818 × 10^−5^ Å, *V*_cell_ = 47.238 Å^3^ and the scale factor *g* = 64 for better visualization of the lines with the used angular resolution of 0.02°. The exact function for intensity simulation was

where the sum runs over all *H* reflections of integer *hkl* indices, π*N* = 100,

and
