# Supplementary material for: Simulation of bright and dark diffuse multiple scattering lines in high-flux synchrotron X-ray experiments
Source: J Appl Crystallogr. 2025 May 31;58(Pt 3):859–68. doi: 10.1107/S1600576725003553 (PMC12135989; doi:10.1107/S1600576725003553)
Supplement: Supplementary file 1 [file j-58-00859-sup1.pdf]

**Supporting information for article:**

# Simulation of bright and dark diffuse multiple scattering lines in high-flux synchrotron X-ray experiments

Maurício B. Estradiote, A. G. A. Nisbet, Rafaela F. S. Penacchio, Marcus A. R. Miranda, Guilherme A. Calligaris, Sérgio L. Morelhão

## 1. ANIMATED GIFS

Simulation of crystal trunction rod (CTR) contributions to diffuse multiple scattering (DMS) lines follows the computational procedure detailed in Appendix C to obtain the S-ring HKL coordinates,

$$\boxed{(\omega,\Phi)_{mn}} \rightarrow \boxed{\boldsymbol{k}^{(c)}_{mn,b}} \rightarrow \boxed{\boldsymbol{k}^{(c)}_{mn,d} = \boldsymbol{k}^{(c)}_{mn,b} - \boldsymbol{Q}^{(c)}} \rightarrow \boxed{\boldsymbol{S}^{(c)}_{mn} = \boldsymbol{k}^{(c)}_{mn,d} - \boldsymbol{k}^{(c)}} \rightarrow \boxed{[\mathrm{h}',\mathrm{k}',\mathrm{l}']_{mn}}.$$

### a. Silicon (001)

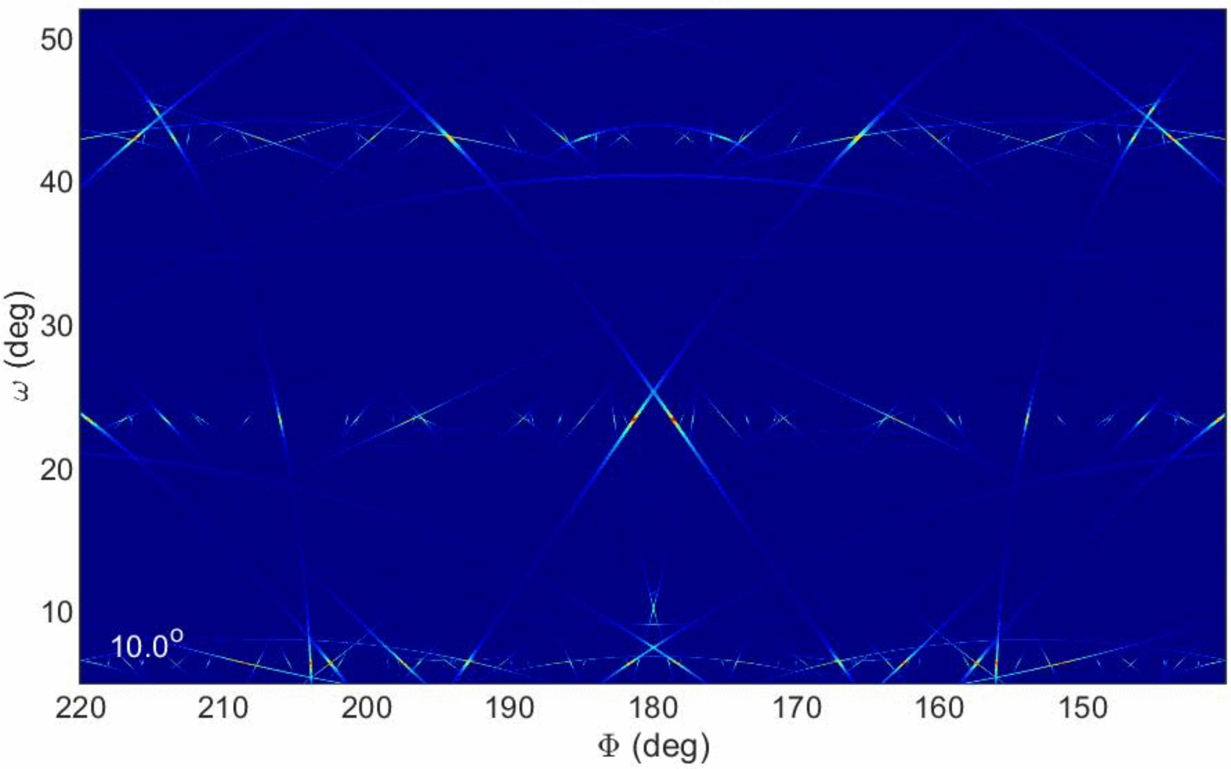


FIG. S1. Animated demonstration of CTR contributions to DMS line intensities in a Si (001) crystal. Vertical scattering geometry. X-ray energy of 8 keV. Incident angle $\omega_0$ varies (value at bottom-left), azimuth fixed at zero ($\Phi_0 = 0$). Line contrast in log scale. Pixel resolution of 0.02°.

For Fig. S1, crystal's reference frame (Appendix A): $\boldsymbol{A} \parallel [001]$ and $\boldsymbol{B} \parallel [1\bar{1}0]$ in vertical scattering geometry ($\chi = 90°$, $\hat{\boldsymbol{\varepsilon}}_0 = \hat{\boldsymbol{\sigma}}$); see Fig. S2 for horizontal scattering geometry ($\chi = 0$, $\hat{\boldsymbol{\varepsilon}}_0 = \hat{\boldsymbol{\pi}}$). The line width scale factor $g = 200$ was used for better visualization of the lines

within the pixel resolution of the solid angle considered in the simulations, and

$$I_{\rm Q}\left([{\rm h}',{\rm k}',{\rm l}']_{mn}\right) = I_0\,|F_{\rm Q}|^2\,\boldsymbol{\varepsilon}\cdot\boldsymbol{\varepsilon}^*\sum_{\rm H}\frac{|F_{\rm H}|^2}{[1+(\pi N_{xy})^2(u^2-u_z^2)]\,[1+(\pi N_z)^2u_z^2]} \tag{S1}$$

stands for the exact function for intensity simulation with $\pi N_{xy} = 1000$, $\pi N_z = 100$, $u^2 = ({\rm h}'-{\rm h})^2 + ({\rm k}'-{\rm k})^2 + ({\rm l}'-{\rm l})^2$, and $u_z^2 = ({\rm l}'-{\rm l})^2$.

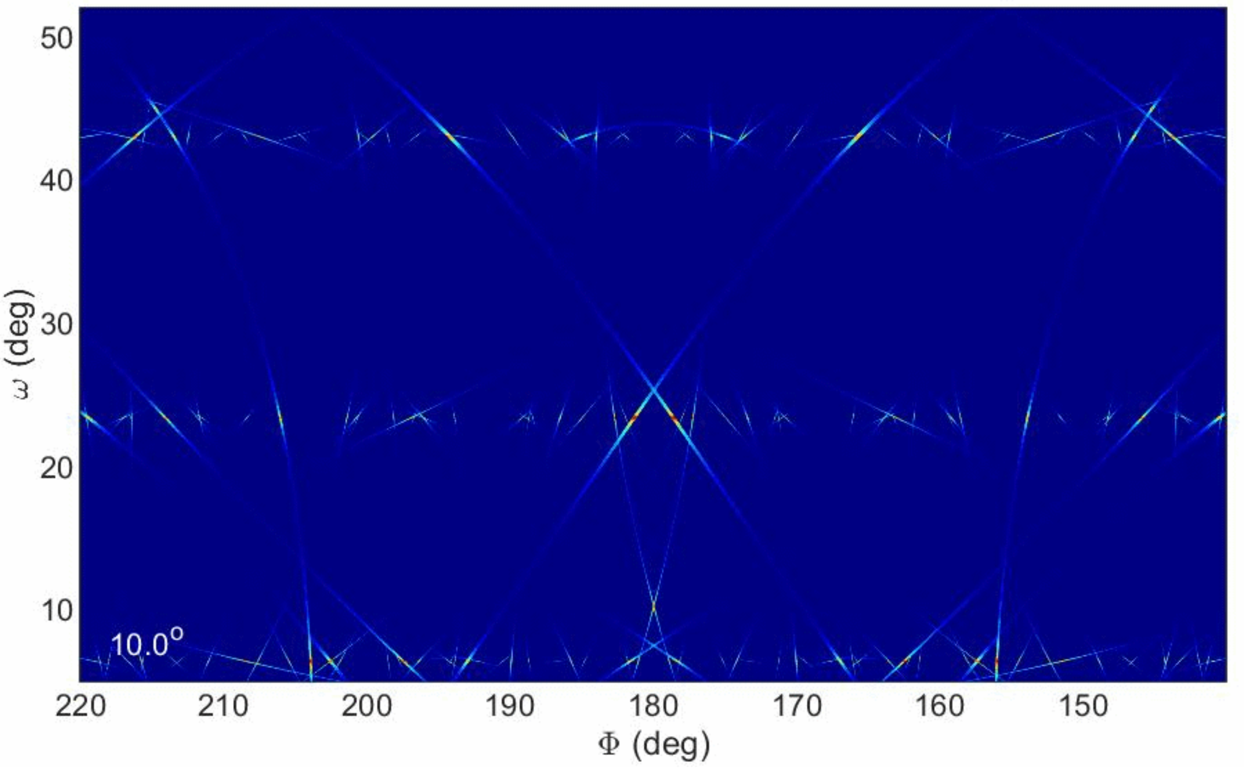


FIG. S2. Animated demonstration of CTR contributions to DMS line intensities in a Si (001) crystal. Horizontal scattering geometry. X-ray energy of 8 keV. Incident angle $\omega_0$ varies (value at bottom-left), azimuth fixed at zero ($\Phi_0 = 0$). Line contrast in log scale. Pixel resolution of 0.02°.

### b. Copper (311)

For Fig. S3, crystal's reference frame (Appendix A): $\boldsymbol{A} \parallel [111]$ and $\boldsymbol{B} \parallel [100]$ in vertical scattering geometry ($\chi = 90°$, $\hat{\boldsymbol{\varepsilon}}_0 = \hat{\boldsymbol{\sigma}}$). The line width scale factor $g = 100$ was used for better visualization of the lines, and the exact function for intensity simulation is the one given in Eq. S1, except that $u_z^2 = \left[3({\rm h}'-{\rm h}) + ({\rm k}'-{\rm k}) + ({\rm l}'-{\rm l})\right]^2/11$ as the CTR is along the surface normal direction [311].

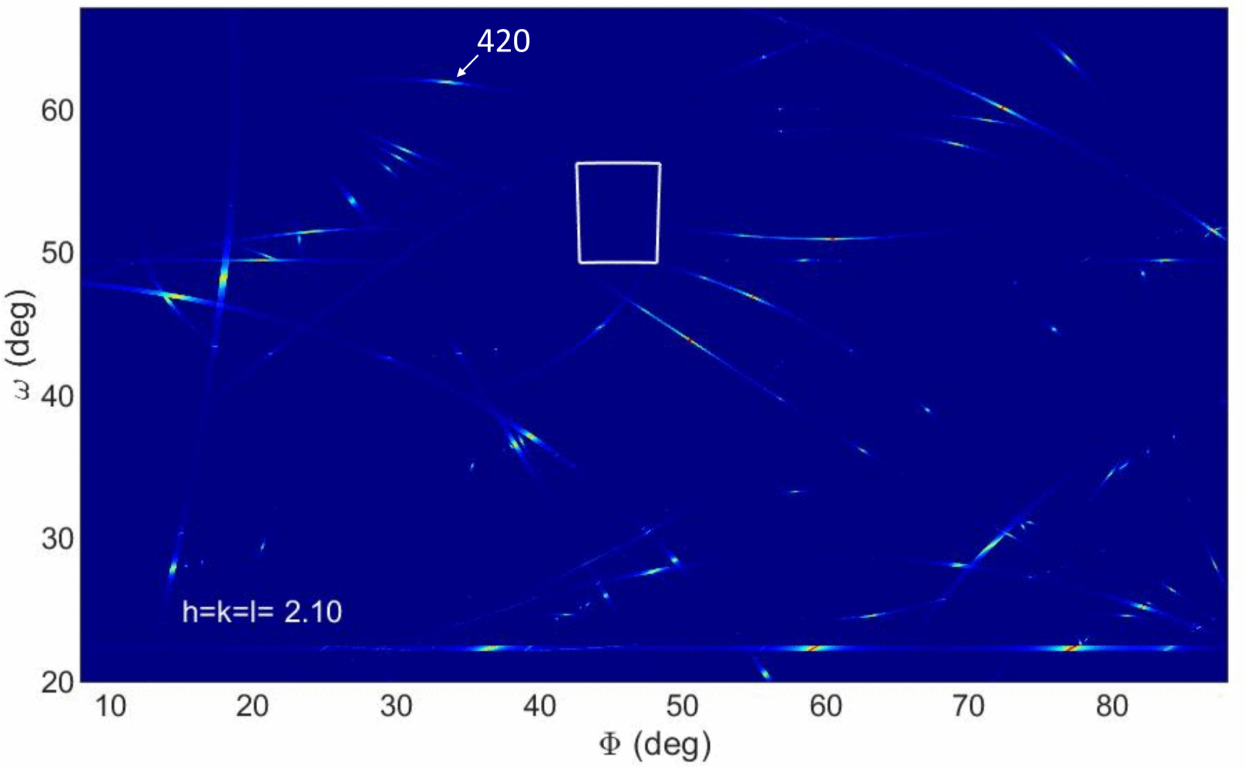


FIG. S3. Animated demonstration of CTR contributions to DMS line intensities in a Cu (311) crystal, as indicated for the 420 line (arrow). Incident angle $\omega_0 = \arcsin\left(\frac{\lambda}{2a}\sqrt{\mathrm{h}^2+\mathrm{k}^2+\mathrm{l}^2}\right)$ varies with the h = k = l value (bottom-left), azimuth fixed at $\Phi_0 = 227.5°$. X-ray energy of 7.82 keV. Line contrast in log scale. Pixel resolution of 0.02°. Position of the 100k detector (white-outlined area) varies with $2\theta_d = 2\omega_0$ while $\varphi_d = 0$ (see Appendix A for details).

The CTR contribution on the 420 DMS line (Fig. S3) shifts to lower $\omega$ values with increasing incidence angle (higher hkl values). It is a further confirmation that the experimentally observed intensity spot on the 420 line in Figs. S4(a)-S4(g) has another cause than the S-ring interaction with the CTR.

## 2. SIMULATION OF EXPERIMENTAL DATA

The simulated images in Figs. S4(h)-S4(n) were obtained by converting the spatial coordinates of pixel $mn$ into $(\omega, \Phi)_{mn}$ angular coordinates in the crystal's reference frame where $\boldsymbol{A} \parallel$ [111] and $\boldsymbol{B} \parallel$ [100], vertical scattering geometry ($\chi = 90°$, $\hat{\boldsymbol{\varepsilon}}_0 = \hat{\boldsymbol{\sigma}}$). For the 100k detector, this conversion uses the parameter vector $P = [565\,\mathrm{mm}, 90°, \omega_0(j), 225.44°, 2\omega_0(j), 0, 35°, 0, 0]$,

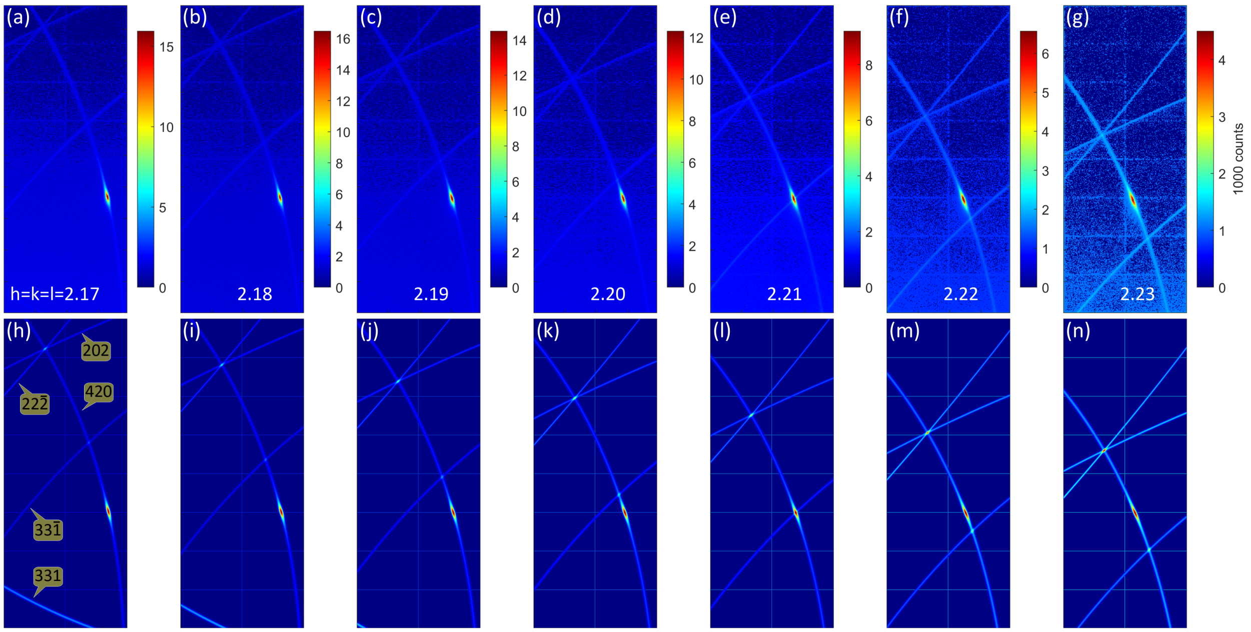


FIG. S4. (a-g) Experimental observation and (f-n) simulation of DMS lines in Cu (311) single crystal with high-flux synchrotron X-rays of 7.82 keV ($\lambda = 1.585486$ Å), $\sigma$-polarization. h = k = l values are indicated (top panels). Simulation is based on isotropic diffuse scattering to light up the DMS lines plus mosaicity at reflection $\bar{2}02$ to account for the behaviour of the intensity spot (red spot) on the 420 DMS line, as described in the main text.

as defined in Appendix A, for which

$$\omega_0(j) \in \arcsin\left(\frac{\lambda\sqrt{3}}{2a}\{2.17,\, 2.18, \ldots,\, 2.23\}\right)$$

with $a = 3.6149$ Å (copper lattice parameter).

The sharpness of DMS lines is adjusted by function

$$B_{mn} = [1 + \mathrm{e}^{+\zeta\,(W_{mn} - \Omega/2)}]^{-1}[1 + \mathrm{e}^{-\zeta\,(W_{mn} + \Omega/2)}]^{-1} \tag{S2}$$

where $W_{mn} = \left(2\boldsymbol{k}^{(c)}_{mn,b} - \boldsymbol{Q}^{(c)}\right) \cdot \boldsymbol{Q}^{(c)}$, $\Omega = \Gamma\,|F_{\mathrm{H}}|$ (Appendix C), and $\zeta$ is the actual sharpness parameter, as shown in Fig. S5. The DMS line intensities are calculated only when $B_{mn}$ is non-zero, practically implemented by using the product of $I_{\mathrm{Q}}\left([\mathrm{h}', \mathrm{k}', \mathrm{l}']_{mn}\right)$ and $B_{mn}$. The sharpness factor of $\zeta = 80/\Omega$ was applied to all the simulations on wide solid angles, Figs. S1, S2, and S3, while $\zeta = 4/\Omega$ was applied to the simulation of experimental data in Figs. S4(h)-S4(n).

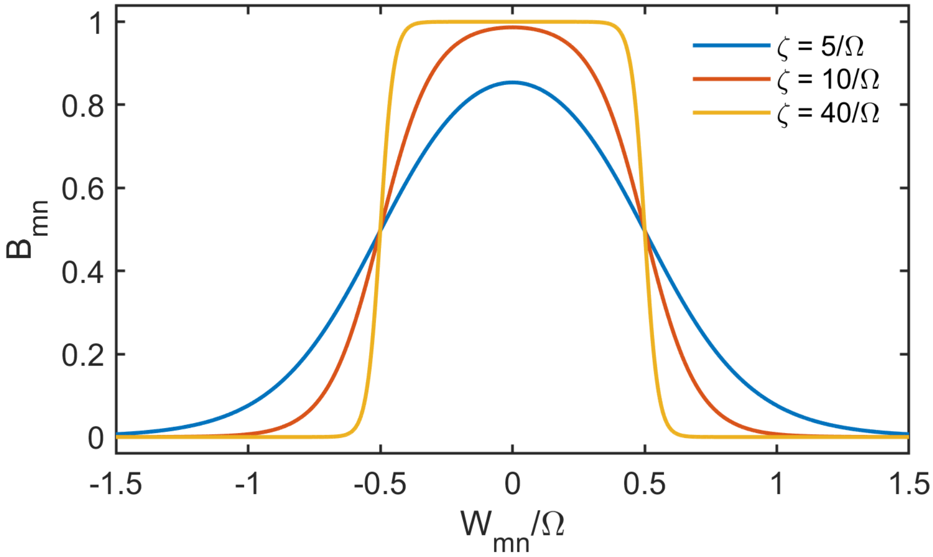


FIG. S5. Sharpness of DMS lines of width $\Omega$ as a function of parameter $\zeta$, Eq. (S2).
