# Supplementary figures and images for: Simulation of bright and dark diffuse multiple scattering lines in high-flux synchrotron X-ray experiments
Source: J Appl Crystallogr. 2025 May 31;58(Pt 3):859–68. doi: 10.1107/S1600576725003553 (PMC12135989; doi:10.1107/S1600576725003553)

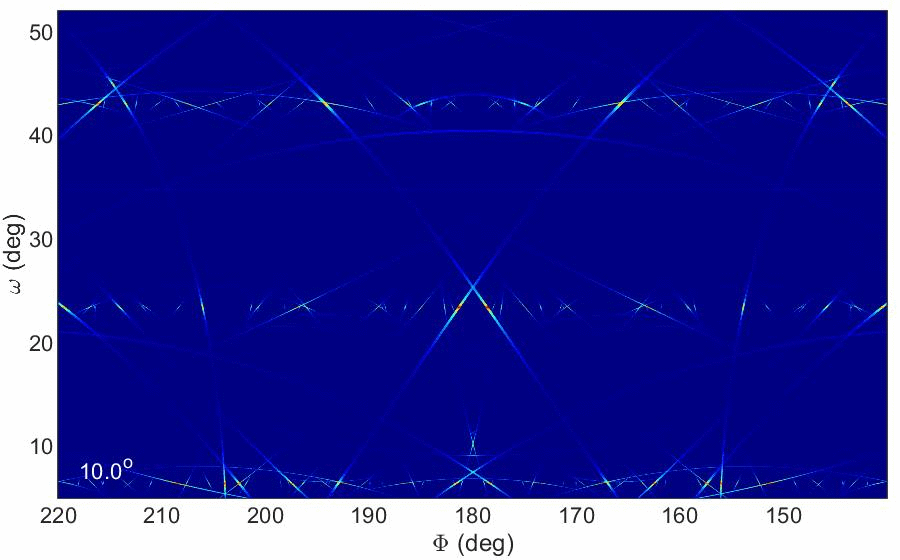

Supplement: Supplementary file 2 [file j-58-00859-sup2.gif]

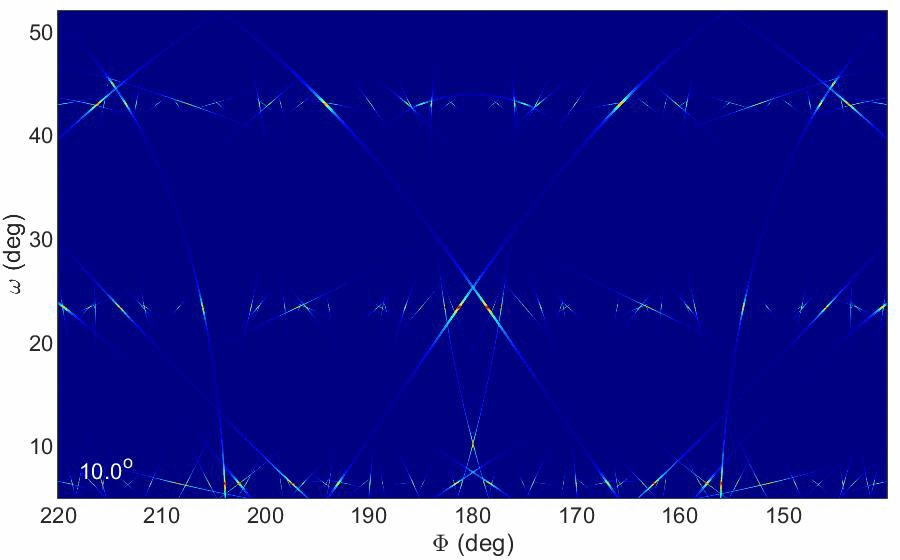

Supplement: Supplementary file 3 [file j-58-00859-sup3.gif]

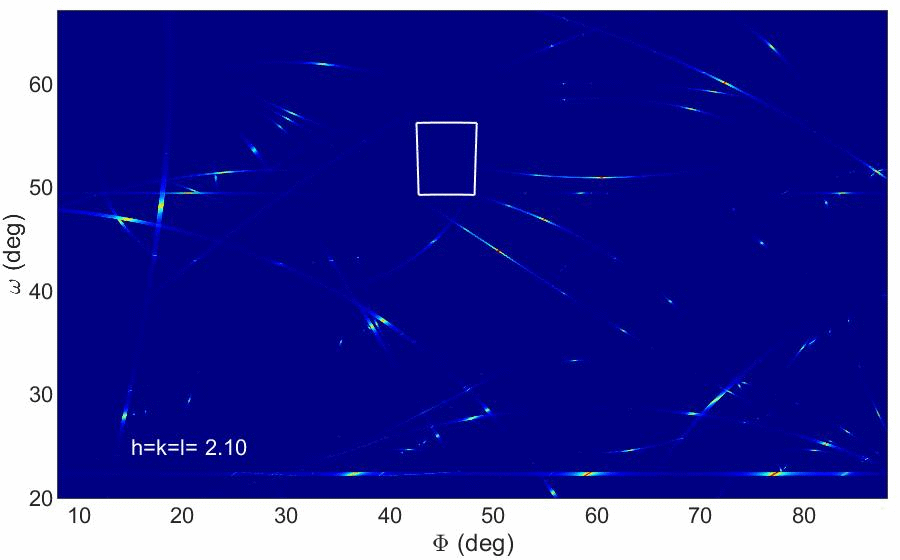

Supplement: Supplementary file 4 [file j-58-00859-sup4.gif]
